# Twelve Tips for Creating an All-Day Writing Retreat for Health Profession Educators: An Immersive, Product-Oriented Learning Experience

**DOI:** 10.15694/mep.2019.000188.1

**Published:** 2019-10-08

**Authors:** Betty Lee Ligon, Remijio Elizondo, Satid Thammasitboon

**Affiliations:** 1 Baylor College of Medicine; 2 Texas Children's Hospital

**Keywords:** scholarship, experiential learning, publishing, authorship, ethics

## Abstract

This article was migrated. The article was marked as recommended.

Health profession educators are keenly aware of the challenges associated with meeting institutional expectations to disseminate their scholarship in forms of publications. In the past, we addressed some of these challenges and described an interactive workshop that we developed from didactic presentations, using educational, psychological, and rhetorical theories. Because we received very positive reviews from workshop participants, we decided to take the next step and develop an all-day writing retreat. In developing this retreat, we spent considerable time preparing in advance; implemented various theories for engaging students and other audiences; and learned lessons along the way. We now offer our insights as tips to help other educators develop a writing retreat.

## Introduction

Most health profession educators (HPEs) are keenly aware of the challenges associated with meeting institutional expectations to disseminate their scholarship in forms of publications ( Sullivan, 2012). We have addressed some of these challenges in the past and described an interactive workshop that we developed from didactic presentations, using educational, psychological, and rhetorical theories ( Ligon, Weinstein and Thammasitboon, 2017) ( Ligon, Turner and Thammasitboon, 2017). Other educators also attest to the value of writing workshops ( Hekelman *et al*., 1995; Steinert *et al*., 2008; Rathmore and Mansoor, 2015). Based on the positive reviews we received from workshop participants, we decided to take the next step and develop an all-day writing retreat in which we translated the material into actual writing experiences; engaged the participants in interactive discussions; and wove together didactic sessions with dedicated times for writing, during which the presenters worked individually with participants. The evaluation scores of the writing retreat were very high ( Table 1), indicating that our approach not only translates into concrete manuscripts that later can be refined for publication, but also engages participants in a unique writing experience.

**Table 1.** An Evalutation of the Retreat

**Facilitators**, overall ranked from poor to excellent, 1 - 5, respectively	Average score
• Were clear about goals and objectives	4.68
• Demonstrated sound communication skills	4.77
• Facilitated interactive discussion/involved all participants	4.72
• Listened and responded effectively to questions	4.72
• Managed time well	4.77
• Used effective tools to achieve objectives (handout/slides)	4.77
**Overall**	**4.74**
**Program Objectives**, ranked from poor to excellent, 1 - 5, respectively	
• Described essential steps for preparing and avoiding pitfalls	4.36
• Described 2-3 practical strategies to enable successful scholarly writing	4.64
• Described 2-3 practical strategies used to enable a successful manuscript submission	4.64
**Overall**	**4.55**
**Activity Value**, ranked from strongly disagree to strongly agree, 1 - 5, respectively, provided information that	
• I will use in my educational activities	4.72
• Met my personal expectations	4.74
• Updated my current knowledge	4.77
**Overall**	**4.74**

In developing this retreat, we spent considerable time preparing in advance, using the expertise of one of the authors who is well-versed in orchestrating different venues; followed our own recommendations for writing effectively, which we discuss below; implemented various theories for engaging students and other audiences ( Kolb and Kolb, 2008; Zhao, 2012; Taylor and Hamdy, 2013); and learned lessons along the way. We now offer our insights as tips to help others who recognize the need to provide faculty and fellows with a full-fledged, all-day writing retreat but are inexperienced in the dynamics of creating and conducting a retreat, if not the actual content.

## Preparation: Behind the Scenes

### Tip 1: Identify an Optimal Learning and Writing Environment

This challenge is part of any experience in writing, and we explained the importance of finding an optimal writing space to the audience as part of the initial didactic presentation. In applying what we teach, we began searching for a locale that would meet at least these two objectives: 1) be sufficient distance from the medical campus that participants could be protected from typical distractions of their jobs ( Shelton *et al.*, 2009) and 2) close enough to the campus that people would not have to drive a long distance in addition to their usual commute. We examined numerous venues before approaching a large church located a short distance from the medical complex. This particular church has a beautiful, spacious facility, audiovisual equipment, and other provisions that it rents for a nominal fee. It also has windows facing a courtyard, which can be utilized along with the room for a respite. It was ideal for our purposes. We realize other institutions may not have such optimal options, but *we recommend finding a setting that achieves at least the first objective of protecting participants from distractions.* Institutions that are located in less congested areas, where participants do not commute long and/or stressful distances, might want to consider a venue located a greater distance from work with an inviting and relaxing environment (e.g., a camp, resort).

### Tip 2: Select a Specific, Designated Time

As in Tip 1, we applied part of what we teach as being crucial to the writing experience, namely *set aside* *an optimal time* for writing. In this instance, we were primarily concerned with a time that would be convenient for most participants. We selected a Friday because most people can get their affairs in order by the end of the week, thereby minimizing distractions, and yet it does not require extra time from home or interfere with other obligations, as a weekend retreat would. Also, Friday opens an extended time to concentrate for those who might want to use the weekend to develop more thoroughly the manuscripts they started in the retreat. Presenters or participants from other cities also can attend the retreat more conveniently on Friday than during the week or on a weekend. Some participants, especially those who travel from other towns, may even use the retreat as part of a weekend get-away in addition to the learning experience. *Given all considerations, we would recommend holding a retreat at the end of the week.*

### Tip 3: Make Provisions for a Smooth-Running Retreat

In addition to identifying an optimal space and designating a specific time, the “behind-the-scenes” logistics are paramount for organization and smooth operation. Each institute will have different requirements, but *we recommend having at least these provisions in place before the retreat starts:* 1) submit the payment information and event forms, including the institution’s particular requirements, and wait for the administration’s approval; 2) pre-determine the budget and create purchase orders in advance for the venue and other expenses, including catering if offered; and 3) request and review at least three quotes for catering, including the menus; estimate additional costs (e.g., supplies, printing, gas, hotel for outside speakers, venue fee) to assist in determining the cost for registration. After these items were completed, we created a registration website that included information about the retreat, fees, and a means to register and pay in advance; once the website was approved, we maintained and monitored it closely. We used the registrations to create a list of participants and relevant data, which we did during the registration process, rather than at the end of registration, when other concerns would be pressing. Promotion of the event required producing brochures, flyers, and e-mail announcements, as well as establishing the audience to be recruited and the person(s) responsible for creating and handling the promotion materials. For the event itself, we had sign-in sheets available, packets to give participants when they arrived (we were able to obtain many items such as small hand sanitizers, pens, and other items at nominal or no cost), and certificates of attendance to distribute at the end of the workshop (names can be entered in advance from the registration). *We recommend implementing at least these steps, using a carefully crafted check-off list ( Table 2), in addition to* *the most important item for organization: a “Time-Line” document, which should be created in advance and have each step in place, from the beginning to the last minute of the event.*

**Table 2.** A sample check-off list for preparing the retreat

Date	Task
	Submit institutional payment form
	Submit institutional approval form
	Obtain costs for outside speaker(s)
	• travel expenses
	• cost for overnight stay (hotel/meals)
	Create purchase orders
	• facility
	• food/catering
	• miscellaneous supplies
	• speaker’s travel expenses (hotel/gas/airfare)
	Solicit bids (including menus) for catering/food
	Establish preliminary budget to determine charges for retreat
	Create a registration website
	Create promotion materials
	• brochure
	• flyer
	• email announcement
	Create items for event itself
	• sign-in sheets
	• packets for handouts
	• name tags

## Development: Content and Organization

### Tip 4: Form a Team of Experts to Cover a Range of Writing Formats

Although the retreat is the fruit of writing presentations and workshops given for many years by one or two of the authors, respectively, we recognized the value of incorporating the expertise of other faculty members in order to offer participants an optimal experience ( Steinert, *et al*., 2008). Hence, we evaluated what information would be valuable to a wide audience of HPEs and decided that, in addition to the information covered on a basic research or review article, we needed to include information on educational innovation and quality improvement. We identified and invited a faculty member who is well-versed in those areas to contribute to the presention of the Materials/Methods and Results sections of a manuscript. We also recognized that the Case Study often is one of the first papers a fellow may write and has specific requirements ( Cohen, 2006). We recruited faculty members who prioritize this teaching and added their work, as a separate presentation, to the overall retreat. Although the presenters all are experts in their specific fields, their contributions extended beyond merely presenting content. Their individual experiences and expertise shared throughout the day reflected different perspectives and resulted in value added to the quality of the sessions. *We encourage engaging as many faculty members from one’s own or other institution(s), as appropriate, to maximize the levels of expertise and scholarship.*

### Tip 5: Implement Theories to Create an Immersive, Product-Oriented Learning Experience

**Figure 1.**
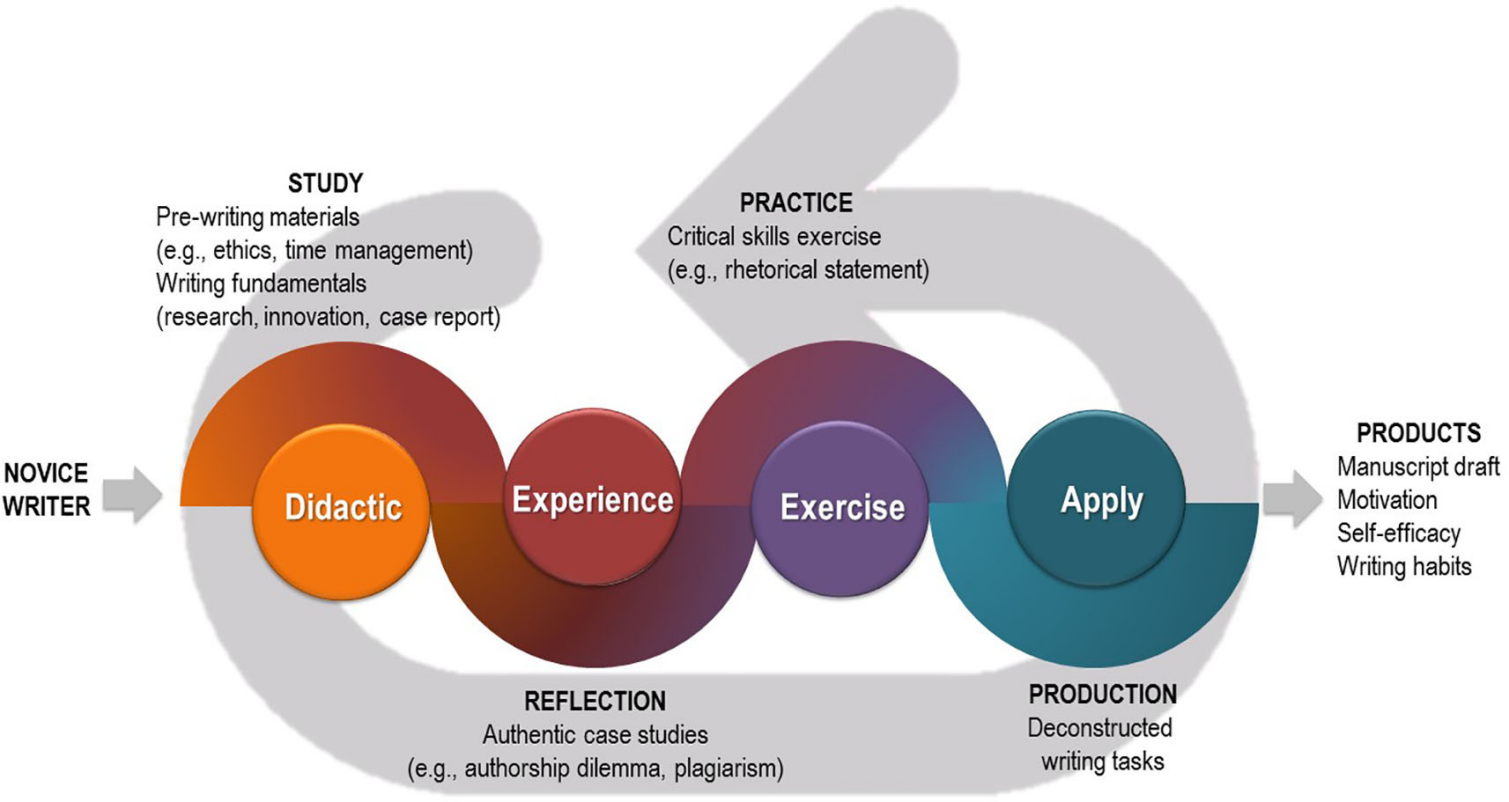
The Conceptual Framework for the Writing Retreat

Many busy HPEs have struggled to write their scholarly work, and might have lost their determination or will over time. Thus, we aimed to create an immersive learning environment ( Blashki, Nichol and Prompramote, 2007; Kolb and Kolb, 2008; de Frietas and Neumann, 2009) that would encourage behavioral and attitudinal shifts from a “can’t do” to “can do,” in one day ( Figure 1).

The strategically sequenced topics allow participants to learn important concepts and skills and progress through writing tasks step-by-step. Each topic comprises four important components (Didactic, Experience, Exercise, and Application). Brief didactic sessions introduce writing fundamentals. Authentic case studies or examples are used for facilitated group discussions and emphasize important concepts. Participants practice certain concepts for immediate feedback prior to applying lessons learned to individual writing tasks.

We concurred that an all-day retreat should be constructed such that each participant would leave with at least a skeleton (more than an outline) of a manuscript. To that end, we determined to present and apply psychological and psychosocial insights related to prewriting ( Boise, 1989, Cloud and Townsend, 1992; McRaven, 2017), followed by individual sessions for each portion of a manuscript, interweaving didactic presentations with authentic writing opportunities. For instance, after we presented the purpose of the introduction and what to include, we immediately allowed time for the participants to work on their introductions while all the presenters circulated around the room to offer help. The didactic content served as a modified “just-in-time” learning experience ( Novak *et al*., 1999; Iannarelli, 2009) that facilitated each portion of the writing task, and thereby enhanced their writing products. Also, by first receiving the didactic teaching and then having time for writing, participants had a break from writing one section before starting to work on the next section. This product-oriented learning ( Zhao, 2012), with shifts from transmitting knowledge to actual applications, was designed to facilitate product-making by inducing self-efficacy in writing. The interweaving of teaching/engaging provides time to absorb information without overload and then opportunity to apply what has been learned, for better retention as well as the practicality of creating a product. *We recommend applying the prewriting portion, even as it is being presented, and then immersing the participants in an actual writing experience after each didactic presentation.*

### Tip 6: Conceptualize and Arrange Topics in a Logical Progression

Although we already had in place most of the didactic material, we made significant rearrangements of the material to fit an all-day retreat format. For example, we presented the information on protecting one’s time, space, and writing experience as an introduction, explaining from psychological theory the advantages of doing so and how we were actually implementing what we were teaching (e.g., we had the participants silence electronic devices and alerts). We then covered the matters of publication ethics (COPE; International Committee of Medical Journal Editors) and other pre-writing concerns ( Keyes, 1995; Kinneavy, 1969), emphasizing how we were implementing these pre-writing steps in their retreat experience. Afterwards, we spent most of the day looking at the different sections of a research paper and then having the participants write that portion while the presenters circulated around the room to mentor or counsel them. Because the Case Study was being presented as a separate entity, we put that presentation near the end of the day, after the participants had written the different portions of a basic research manuscript. In the final session, we demonstrated the value of proofing and editing, using comical illustrations to illustrate the impact of accurate punctuation, syntax, and other concerns. We deliberately saved this portion until the end of the day because, despite the importance of these considerations, we could present them in a light and humorous fashion to presenters, who were tired by that time. *Given the feedback, we would recommend and will use again this progression of presentation* *and this type of interweaving, as it allows for focus on individual portions of manuscripts and opportunities for application.*

### Tip 7: Determine the Length of Time to Allot for Each Writing Task

Breaking the sessions into workable compartments of time was quite a challenge, especially when we decided to expand the Material/Methods and Results sections to incorporate education innovation and quality improvement. We wanted to ensure that the participants had sufficient writing time to produce an actual manuscript rough draft, not just interactive practice. We allowed approximately 20 - 30 minutes for each writing task, and then we allocated the time that we would have for the accompanying didactic portion. We divided the day into exact lengths of time (e.g., 8:15 - 8:45) and put the “finish time” for each presentation (in this case, 8:45) on a schedule given to each presenter and highlighted the finish time. We allowed flex time of 5 minutes in the morning and 10 minutes in the afternoon. By being this specific, and with the cooperation of all the presenters, we were able to stay exactly on schedule and to finish on time without jeopardizing any portion of the didactic or writing components. We purposefully allowed the lunch time to be long enough that participants had plenty of time to converse about their shared experience and some “struggles” in writing and publishing their scholarly work. *We highly recommend using a similar system, as presenting so much information can interfere with allowing sufficient time for writing.*

## Delivery: An Engaged, Authentic Experience

### Tip 8: Embed Exercises Conducive to Immediate Application

Much of the introductory information is didactic, so we decided to engage the participants initially in the ethics of who does or does not qualify as an author by having them list all *potential* authors in space designated in the workbook (see Tip 10); we then presented the criteria for authorship according to COPE and other institutions, after which we had the participants review their lists and check the authors who actually qualify. This exercise allowed them immediate engagement with the material presented. The next such writing exercise involved the rhetorical statement ( Kinneavy, 1969). After explaining the importance of *stating* the value of their research or work, rather than merely assuming the reader would recognize it, we had participants write one or two sentences stating the importance of their results. They shared these statements with their peers for input and improvements, as well as immediate recognition of what might be lacking, and several participants shared their statements with the entire audience. Their “rhetorical statements” were entered in the workbook on the appropriate page and designated for later use in different portions of their manuscripts. This exercise opened opportunities for partipants to engage their peers in meaningful conversations. All of the other writing experiences pertained to the various portions of their manuscripts, which they wrote after we presented the didactic material (See Tip 5). *We highly recommend identifying portions of the knowledge content that can be discussed and applied immediately so that the audience begins to participate in essentials of writing the manuscript, at the beginning of the retreat.*

### Tip 9: Use Authentic Case Studies to Highlight Critical Learning Points

Some concerns of writing require a special emphasis in order to obviate or circumvent serious potential consequences but might not be apparent within the participants’ current projects or part of their past writing experiences. Those topics include ethical principles such as plagiarism ( Das and Panjabi, 2011), sketchy literature research, and the dilemma of determining authors’ rankings ( Strange, 2008). Rather than presenting these matters as principles only, we used actual case studies to highlight their serious nature and to engage the participants in conversations, attempting to solicit as many actual examples or experiences from the participants as possible. Additionally, we presented authentic case situations or studies from our own experiences to highlight some potential pitfalls they might encounter in scholarly writing. Participants discussed and/or debated the cases, after which the facilitators summarized critical learning points. Having a broad range of speakers/facilitators proved to be especially useful in these presentations, as individual experts shared invaluable experiences from different viewpoints and settings. Our participants invariably informed us about how much they learned from these discussions, and *we recommend involving them in this fashion for both development of intellectual acumen and peer engagement that allows them to express concerns, ask questions, and share their own experiences.*

### Tip 10: Provide an All-In-One Learning Resource for the Retreat and Beyond

A well-received aspect of our workshops and presentations has been the accompanying bound workbook that we provide. For the retreat, we offered a similar document, which synthesizes ALL the content from the slides, which included the corresponding page numbers so the participants could follow along easily and make notes. Because most of the participants were using laptop computers to write, we provided only minimal space in the workbook for writing, but we did have specific write-in spaces for the discussions on qualifications of authors and the rhetorical statement (see Tip 8). The workbook also had supplementary information. For instance, in addition to having the slides’ content on plagiarism, we included articles that address plagiarism and instances of actual plagiarism that resulted in dire consequences for the perpetrators (e.g., Chalmers, 2006), as well as an article on predatory journals ( Beall, 2013) and an article on writing a case study ( Cohen, 2006). In the appendices, we included templates for different types of articles. The presenters of the case study also provided a workbook, complete with an entire listing of “predator journals.” Many participants commented about their appreciation for having take-home documents to reinforce what the retreat offered. *We recommend providing materials that expand the information covered in the retreat, both to substantiate the materials presented in the retreat and to enhance the learners’ knowledge.*

## Improvement: The Future

### Tip 11: Gather Program Evaluations for Ongoing Improvements

Although evaluation forms are rather standard, we ascertained to gather information pertaining to both the *process* and the *product* of the retreat. Hence, we identified six items to evaluate the effectiveness of the facilitators, program content, and logistics, on a scale of 0 - 5, with 0 being the least effective and five being the most effective. Scores for facilitators ranged from 4.7 to 4.8 (overall, 4.73); for meeting program objectives, we received 4.36 to 4.64 (overall, 4.55); and for the value of the retreat, we received scores ranging from 4.7 to 4.8. These scores helped us identify what to continue doing and what to correct or enhance. We asked two questions for feedback. One question was: *“What was most helpful to you in this teaching session?”* The answers included: “Independent writing time with supervision”; “The workbooks to take home with lecture points”; “Breaking down the manuscript into concrete and clear pieces”; and “Hands-on time with manuscript.” The second question was: *“What additions or changes would you make?”* The answers included: “I expected more writing/revision assistance & individualize [sic] help. 5 experts and >20 learner [sic] made that expectation unrealistic”; “More help for case report writing”; “Similar session on figure/chart building. Extensions cords; square tables”; and “Discussion of organizing your lit review.” We also evaluated whether participants had executed what they learned into actions (i.e., the product) with a follow-up survey. At four months after the retreat, 25% of the participants reported they were about to submit a manuscript, and 17% had almost finished the first draft. The remainders were working on their drafts. We plan to follow-up with them and offer editorial consultations periodically. *Because of unique usefulness for the future that these evaluations provide, we encourage the development of evaluations or feedback documents in order to always be improving.*

### Tip 12: Make Plans for Going Forward

We are always open to ways to improve our presentations, and particularly this retreat. We learned from participants’ feedback, both positive and negative comments. Although the ratio of “experts” to participants was, indeed 5:20, as noted, we circulated and mentored individuals who needed help, and someone was always available to answer questions. Because it was a writing retreat, rather than a mentoring session per se, and only one participant expressed this concern, we are pleased with the results. We will probably continue to use round, rather than “square,” tables because they have many advantages. The request for extension cords is noted, as is providing a more in-depth discussion of organizing the literature review. One of our Vice Chairs, after reading the evaluations, offered another suggestion that we definitely will incorporate into the next retreat, which is to offer participants the opportunity to send manuscripts in advance for editing and then meet with them individually to discuss the edits. This idea could also form the basis for a separate workshop or retreat with different objectives *. As we consider how to move forward, we encourage our readers to do the same with the feedback they receive.*

## Conclusion

Given the high scores in the feedback from our retreat, coupled with the literature, we are more convinced than ever of the value and, indeed, the importance of offering HPEs a retreat - both from the demands of their jobs and for an opportunity to focus exclusively on writing. The tips we offer here are intended to help other institutions have equally successful retreats and avoid different pitfalls that we encountered along the way when giving workshops and presentations. We plan to implement them again in the future, with the hope of broadening our audience to other institutions in the region.

## Take Home Messages

• Investing time in pre-retreat preparations, including establishing a minute-by-minute schedule, is essential to having an organized and smooth-running event.
• Interweaving didactic presentations with designated time for actual writing helps participants concentrate on specific portions of the manuscript and apply immediately what they have learned.
• Engaging participants in open discussions with several expert presenters enhances their experiences and broadens their knowledge and skills base.
• Having presenters with various areas of expertise serve as one-to-one mentors while the participants are writing offers efficient means of motivating and building one’s self-efficacy in the writing process.

## Notes On Contributors

Betty Lee Ligon, PhD, MA (English, Rhetoric), MA (English, Modern British Literature), MAR (Theology), is department medical writer, editor, and educator, and a core faculty member of the Center for Research, Innovation, and Scholarship in Medical Education, Department of Pediatrics, Baylor College of Medicine/Texas Children’s Hospital, Houston, Texas. She taught English literature and writing for more than 25 years at a private university, where she created the Professional Writing Program.

Remijio Elizondo is senior administrator and program manager for the Center for Research, Innovation and Scholarship in Medical Education, Department of Pediatrics, Baylor College of Medicine/Texas Children’s Hospital, Houston, Texas.

Satid Thammasitboon, MD, MHPE, is Associate Professor of Pediatrics, Baylor College of Medicine, and the Director for the Center for Research, Innovation and Scholarship in Medical Education, Department of Pediatrics, Baylor College of Medicine/Texas Children’s Hospital, Houston, Texas.
