# Supplementary material for: Twelve Tips for Creating an All-Day Writing Retreat for Health Profession Educators: An Immersive, Product-Oriented Learning Experience
Source: MedEdPublish (2016). 2019 Oct 8;8:188. [Version 1] doi: 10.15694/mep.2019.000188.1 (PMC10712479; doi:10.15694/mep.2019.000188.1)
Supplement: Peer review history

# Reviewer report: Reviewer response for version 1

**DOI:** 10.21956/mep.19878.r30059

**Published:** 2019-10-29

**Authors:** Alexander Woywodt

**Affiliations:** 1 Lancashire Teaching Hospitals NHS Foundation Trust

This review has been migrated. The reviewer awarded 4 stars out of 5 I found this an interesting paper to read. Turning enthusiasm and ideas into publications is a challenge that many institutions face. There is reason to believe that many good ideas and concepts do not make it into publications because authors are daunted by the challenge, don't know where to start or how to pitch their ideas, or are too busy with non-academic work. I think this article provides useful experience and a toolkit for colleagues who are thinking of providing a similar workshop. I do wonder what support is available after the retreat and the authors report that they provide help afterwards. I think the approach would be further enhanced if the retreat would be combined with some sort of structured mentoring afterwards.

Reviewer Expertise:

NA

No decision status is available

# Reviewer report: Reviewer response for version 1

**DOI:** 10.21956/mep.19878.r30062

**Published:** 2019-10-16

**Authors:** Dujeepa D. Samarasekera

**Affiliations:** 1 National University of Singapore

This review has been migrated. The reviewer awarded 3 stars out of 5 Useful tips for faculty who are involved in developing writing retreats. Having organised a few of these retreats, the Tip 11 and especially the Tip 12 are very useful. It would have been good to include a Tip or Tips on how best to follow-up in developing the draft (skeleton) to a full manuscript. This could include how the organisers would be able to provide further support to the participants. Another could be on how best to leverage on school/institution level support for organising and executing these activities. Overall, I enjoyed reading the article. Thank you.

Reviewer Expertise:

NA

No decision status is available

# Reviewer report: Reviewer response for version 1

**DOI:** 10.21956/mep.19878.r30060

**Published:** 2019-10-09

**Authors:** Michelle McLean

**Affiliations:** 1 Bond University

This review has been migrated. The reviewer awarded 4 stars out of 5 Thank you for these 12 tips. They will be most useful for those charged with FD in this area. I think getting away from the campus in a conducive setting is important. I may have missed this in the text. Was the participation voluntary? Did the writing involving individuals or teams of collaborators? I guess these queries are more about who attends as this would impact on the organisation such as having space for discussion without disturbing others.

Reviewer Expertise:

NA

No decision status is available

# Reviewer report: Reviewer response for version 1

**DOI:** 10.21956/mep.19878.r30061

**Published:** 2019-10-09

**Authors:** Mzwandile Mabhala

**Affiliations:** 1 University of Chester

This review has been migrated. The reviewer awarded 3 stars out of 5 This article would be useful for those who have a responsibility for organising continuing professional development.

Reviewer Expertise:

NA

No decision status is available
